# PROSPECTIVE CONTROLLED STUDY OF SPINAL SURGERY VERSUS PHYSICAL CAPACITY

**DOI:** 10.1590/1413-785220233101e259011

**Published:** 2023-04-17

**Authors:** Vam Charly Pereira Araújo Almeida, Hector Figueiredo Felix, Fernanda Andrea Minutti Navarro, Ana Fatima Salles, Carolinne Nascimento de Oliveira, Luiz Claudio Lacerda Rodrigues

**Affiliations:** 1 Hospital Santa Marcelina, Orthopedics and Traumatology Service, São Paulo, SP, Brazil.; 2 Hospital Santa Marcelina, Spinal Pathologies Group, São Paulo, SP, Brazil.; 3 Universidade Federal de São Paulo, São Paulo, SP, Brazil.; 4 Faculdade de Medicina Santa Marcelina, São Paulo, SP, Brazil.; 5 Faculdade de Medicina Santa Marcelina, Santa Marcelina Hospital, Spinal Pathologies Group, São Paulo, SP, Brazil.

**Keywords:** Orthopedic Surgery, Osteoarthritis, Spine, Exercise Test, Cirurgia Ortopédica, Osteoartrite da Coluna Vertebral, Teste de Esforço

## Abstract

**Objectives:**

Assess whether the spine surgical approach for degenerative diseases can influence the physical capacity of patients and its correlation with cardiorespiratory function.

**Methods:**

A prospective study was conducted on 9 patients of both genders, aged between eighteen and sixty, scheduled for spinal surgery for degenerative disease in the lumbar segment. Patients underwent treadmill stress test two times, fifteen days before and sixty days after the surgery. A cardiologist performed the test according to the Bruce protocol with a progressive increase in incline and speed.

**Results:**

There were no statistically significant differences between pre- and postoperative assessments for the parameters evaluated in the treadmill stress test. Forty-four percent of patients needed to interrupt the test postoperatively due to dyspnea (p=0.023).

**Conclusion:**

The improvement obtained with spinal surgery does not have statistically significant relevance in tiredness, pain, and fatigue in the lower limbs and low back pain. Some patients could not complete the examination after surgery due to poor physical conditioning, and it was necessary to interrupt the examination due to dyspnea. ***Level of Evidence II; Lesser quality RCT (eg, < 80% followup, no blinding, or improper randomization).***

## RESUMO

**Objetivo:**

Avaliar se a abordagem cirúrgica da coluna vertebral para doenças degenerativas pode influenciar a capacidade física dos pacientes e sua correlação com a função cardiorrespiratória.

**Métodos:**

Um estudo prospectivo foi realizado em 9 pacientes, de ambos os sexos, com idade entre 18 e 60 anos, programados para cirurgia de coluna vertebral para doenças degenerativas no segmento lombar. Os pacientes foram submetidos a teste de esforço em esteira duas vezes, quinze dias antes do procedimento cirúrgico e sessenta dias após a cirurgia. O teste foi realizado por um cardiologista de acordo com o protocolo Bruce com aumento progressivo de inclinação e velocidade.

**Resultados:**

Não houve diferenças estatisticamente significativas entre as avaliações pré e pós-operatórias para os parâmetros avaliados no teste de esforço em esteira. Quarenta e quatro por cento dos pacientes precisaram interromper o teste no pós-operatório devido à dispnéia (p=0,023).

**Conclusão:**

A melhora obtida com a cirurgia da coluna vertebral não apresenta impactos estatísticos no cansaço, dor e fadiga relativos aos membros inferiores e dores lombares baixas. Alguns pacientes não puderam completar o exame após a cirurgia devido ao mau condicionamento físico, exigindo a interrupção do exame devido à dispnéia. ***Nível de Evidência II; ECRC de menor qualidade (por exemplo, < 80% de acompanhamento, sem mascaramento do código de randomização ou randomização inadequada).***

## INTRODUCTION

The search for chronic low back pain treatment is growing.^1^ Low back pain is considered chronic if lasts longer than six months. It is estimated that 70 to 85% of the world's population will experience back pain at some point in their lives.^2^ In Brazil about ten million of Brazilians suffer with disabilities caused by low back pain,^3^ which is considered a public health problem.^4^ LBP can be caused by several factors, such as inflammation, muscle weakness and degenerative diseases. Disc degeneration is related to environmental effects, injuries caused by trauma, smoking, atherosclerosis and the natural aging.^5^ The lumbar spine is divided according to its functional capacity; in anterior, middle, and posterior. The anterior portion of spine is made up of the intervertebral discs and vertebral bodies. The middle portion consists of the spinal canal and pedicles. The posterior portion is responsible for directing the functional units in the movements of anterior/lateral flexion, extension and rotation.^6^ The intervertebral disc works as a hydraulic system, absorbing shock and distributing the load evenly.^7^ Thus, the amount of water in its composition it is proportional to its absorption capacity.^8^ Interverterbal disc collagen production occurs in three phases: the initial one, where there is abundant collagen production; the maturation phase, which maintains collagen renewal; and, finally, the degenerative phase, where there is a decrease in renewal.^9^ As it is an avascular structure, the nutrition of the disc occurs by diffusion.^10^ Anatomically described, the soluble regulators of cell function indicate the aging and senescence of the disc - which consists of a nucleus pulposus surrounded by an annulus fibrosus. The nucleus pulposus acts allows the vertebral disc to withstand forces of compression and torsion, permitting the liquid exchange between capillaries and vertebral discs and the axis of vertical movement between two vertebrae. The fibrous annulus, on the other hand, participates in the stabilization and movement of the vertebral bodies and loads dampening.^11^ However, with aging, its biomechanical efficiency starts to decrease due to dehydration.^12,13^ The nucleus pulposus progressively loses its capacity to hold water and the annulus fibrosus begins to lose its elasticity, demonstrating the degradation of the nutrition mechanism and then resulting in disc degeneration.^14^ New studies show that the main factors influencing healthy and degenerated intervertebral disc are: mechanical load, genetic influences, diffusion of nutrients and oxygen through the intervertebral disc matrix.^14^

The treatment of chronic low back pain, among its many causes, includes several therapeutic options, from conservative treatment to surgical intervention.^6^ In this study, we sought to assess the physical capacity of patients undergoing surgical treatment of the spine through the treadmill stress test. In addition to diagnosing cardiovascular diseases, the treadmill stress test also determines prognosis and assesses therapeutic responses to exercise tolerance, in addition to being a test considered to be of low risk. The patient is submitted to a programmed physical effort, which allows the assessment of their clinical, electrocardiographic, hemodynamic, metabolic, autonomic and ventilatory responses. The test will enable the evaluation of the therapeutic interventions results and the patient's actual physical conditions.^15^

The aim of this study was to assess whether the spine surgical approach for degenerative diseases can influence the physical capacity of patients, correlating it with cardiorespiratory function.

## METHODS

This study was approved by the research ethics committee and (number 2.904.787/2018) was carried out in accordance with the 1995 Declaration of Helsinki in the period from May 2019 to May 2020. Nine patients of both genders, aged between eighteen and sixty years, scheduled for spinal surgery for degenerative disease in the lumbar segment were included.

Patients with neuromuscular diseases, history of cardiovascular diseases, spine fractures, any sequelae on spine, pelvis and lower limbs diseases were excluded due to limitations in performing the treadmill stress test.

A prospective study was carried out, with qualitative and quantitative data collection. Patients were submitted to the exercise test in two moments, which would be done fifteen days before the surgical procedure and sixty days after the surgery.

The test was performed by a cardiologist according to the Bruce protocol with a progressive increment in inclination and speed, to assess functional capacity.^9^ For this purpose, a treadmill was used for thirty minutes, and vital signs such as blood pressure, respiratory and heart rate were measured.

Data collection began after approval by the Research Ethics Committee and the signing of the free and informed consent form by the volunteers.

A significance level of 0.05 (5%) was defined for this work. We chose to use non-parametric tests, as the dataset has a low sampling. Pre and post operative tests were compared using the Wilcoxon test, as the data are paired.

## RESULTS

Nine patients were included, 6 females and 3 males. The minimum age was 18 years and the maximum age was 62 years with an average of 45 years.

All patients included had the degenerative condition of the lumbar spine as their underlying disease, and all underwent spinal arthrodesis with instrumentation of up to 3 levels.

When evaluating the associated comorbidities, we considered as obesity patients those with a body mass index (BMI) above 35 and overweight BMI ranging from 28 to 34.9. The distribution of comorbidities was presented in Table 1, remember that comorbidities may overlap, because patients may have more than one associated disease. The results of the pre and postoperative test evaluation are presented in Table 2. There were no statistically significant differences between pre and postoperative assessments for the parameters evaluated in the treadmill stress test.

**Table 1:** Comorbidities distribution.

Comorbidities	N(%)
Obesity	4(44,4)
Smoker	2(22,2)
Ex-smoker	3(33,3)
Arterial hypertension	2(22,2)
Oerweight	2(22,2)
Dyslipidemia	1(11,1)

**Table 2:** Comparison between pre and postoperative for treadmill stress testing.

	Moments	Mean	Median	Standard deviation	Minimum	Maximum	IC	P-valor
Weight (kg)	Pre-Operative	72.1	66	15.6	46	95	10.2	0.513
	Post-Operative	72.8	69	14.7	47	99.4	9.6	
Speed (mph)	Pre-Operative	3.29	3.40	0.52	2.50	4.20	0.34	0.196
	Post-Operative	2.83	3.40	1.29	1.20	4.20	0.84	
Distance (miles)	Pre-Operative	0.299	0.300	0.124	0.110	0.460	0.081	0.374
	Post-Operative	0.261	0.230	0.201	0.010	0.520	0.131	
Interruption time (m:sec)	Pre-Operative	07:40	07:38	02:41	03:30	11:59	01:45	0.214
	Post-Operative	06:14	06:16	04:09	00:30	10:59	02:43	

When we analyzed the reason for the interruption of the test (Table 3), we observed a statistically significant difference (p = 0.023) in one of the reasons for interruption. Forty four percent of the patients needed to interrupt the postoperative test due to dyspnea that not observed in the preoperative evaluation.

**Table 3:** Comparison between the different reasons for interruption of the exam.

Reasons for interruption	Pre-Operative	Post-Operative	P-value
	N (%)	N(%)	
Tiredness	2(22.2)	1(11.1)	0,527
Pain in lower limbs	5(55.6)	4(44.4)	0,637
Fatigue in the lower limbs	1(11.1)	0(0.0)	0,303
Low back pain	1(11.1)	0(0.0)	0,303
Dyspnea	0(0.0)	4(44.4)	0,023 *

* statistically significant p value.

## DISCUSSION

This study evaluated the surgical approach in the treatment of degenerative disease of the spine, with a treadmill stress test performed in the preoperative and postoperative periods (3-month). The mean age of patients was 44.6 years, with a low variability, keeping the data homogeneous and in accordance with the previous literature.^16,17,18^ There was no difference regarding gender distribution, even with the prevalence of female with 66.7% and male with 33.3% (p-value 0.157). This prevalence of female gender was found in several literature review papers for chronic low back pain.^19^ The obesity was the most recurrent comorbidity in this study, with 44.4%, and it may be one of the variables with the most interference in the postoperative evaluation. Patients had an increase in body weight, which may be due to improved pain, mood, and reduced use of oral medications, which could lead to an improvement in gastrointestinal feelings. In several papers, obesity has been listed as an important factor in etiology and pain. One of these studies was carried out with an epidemiological profile of Brazil.^19^

In the comparison pre and postoperative for the treadmill stress tests, our aim was to evaluate the patient's physiological changes after the surgery improvement in the low back pain and the limitation it could be causing. However, it was not significant between these two moments. No previous similar study was found in literature. The moments (pre- and postoperative) and the reasons of treadmill stress test interruption were evaluated. It was found that there is a statistical difference between the moments for the distribution of dyspnea, where the percentage raised from 0.0% in the preoperative period (p = 0.023) to more than 44% in the postoperative period, which led us to think that even with the improvement in pain and functional limitation, obesity may influence the dyspnea, since even some patients being smokers or former smokers, they did not present preoperative dyspnea. We did not find papers to make a comparison with the data obtained in our study.

This study presents us with some questions and directs us to new variables to be observed in the surgical treatment of the spine, emphasizing the importance of metabolic disorders and obesity, for example, not only as triggering factors, but also as harmful factors in the intra and postoperative. Relevant information was given to patients, such as problems encountered in the postoperative period. Aiming to improve clinical conditions and quality of life, trained multidisciplinary professionals such as nutritionists, psychologists and physiotherapists were suggested. We can also think that dyspnea may not have been observed, as the mechanical disorder of chronic low back pain would not allow this patient to make great efforts, which may not allow an activity that would cause dyspnea. The removal of this factor associated with the time of postoperative convalescence could add another factor not analyzed. Perhaps the need for earlier and more intense rehabilitation after surgery, after removing the mechanical factor, could be the best option to improve this limiting clinical condition. Some limitations of our study must be presented – small sample and short follow-up - however, it could provide us with new possibilities for study, leading to future research.

## CONCLUSION

Improvement achieved with spinal surgery does not have a statistically significant in tiredness, pain, and fatigue in the lower limbs in patients with chronic low back pain.

Regarding patients who were not able to complete the treadmill stress test due to physical limitations after surgery, this probably occur due to poor physical conditioning, being necessary to interrupt the test due to dyspnea.
